# External Dacryocystorhinostomy: Characteristics and Surgical Outcomes in Patients with and without Previous Dacryocystitis

**DOI:** 10.1155/2013/287524

**Published:** 2013-12-18

**Authors:** Gilad Rabina, Shani Golan, Meira Neudorfer, Igal Leibovitch

**Affiliations:** Oculoplastic and Orbital Institute, Department of Ophthalmology, Tel Aviv Sourasky Medical Center, Tel Aviv University, 6 Weizman Street, 64239 Tel Aviv, Israel

## Abstract

*Objective*. To compare pre- and postoperative characteristics and surgical success rates of patients with and without previous episodes of dacryocystitis, who underwent external dacryocystorhinostomy (DCR) for nasolacrimal duct obstruction (NLDO). *Methods*. The medical files of all patients who underwent external DCR between 2006 and 2011 in our institution were reviewed. The retrieved data of patients with and without previous episodes of dacryocystitis were compared. Surgical success was determined by postoperative followup of at least 6 months. *Results*. A total of 185 patients with NLDO underwent external DCR of whom 152 (100 females and 52 males, mean age 67 ± 15 years) met the inclusion criteria. Sixty had previous episodes of dacryocystitis and 92 did not. Left-side obstruction was more common than right-side obstruction among patients with previous episodes of dacryocystitis (48.3% versus 31.7%, resp., *P* = 0.031). Glaucoma patients were significantly more likely to develop dacryocystitis than patients without glaucoma (*P* = 0.002). The success rate of external DCR was 94.4% for patients with previous episodes of dacryocystitis and 86.7% for patients without (*P* = 0.337). *Conclusions*. The surgical outcomes of external DCR in patients with or without a previous episode of dacryocystitis were similar. Patients with glaucoma and NLDO had a significantly higher risk of developing dacryocystitis.

## 1. Introduction

External or endoscopic dacryocystorhinostomy (DCR) is the standard treatment for nasolacrimal duct obstruction (NLDO), with or without a previous history of dacryocystitis. The procedure gained popularity due to its efficacy and relatively low rate of complications. The external approach is made through a skin incision near the lacrimal sac and the endoscopic approach is made through the nasal cavity with the aid of a nasal endoscope [1]. The primary benefits of the endoscopic approach are the lack of skin scarring (there are no skin incisions) and quicker healing time, and the primary benefits for the external approach are a faster and easier surgery to perform.

The surgical success rate was found to correlate with the duration of obstruction and patient's age; the surgical success rate was lower when the obstruction lasted longer and the patient was younger [2]. The success rates of the endoscopic approach in NLDO are similar to those of the external approach and range between 80% and 96% [3–11]. This variability in success rates is likely due to differences in surgical techniques, patient demographics, and lack of standardized outcome measures.

Only a few earlier studies examined the effect of past dacryocystitis on the success rates of external DCR. The success rates in those studies were lower than studies who examined DCR without dacryocystitis. Their reported success rates range from 69% to 88% [12, 13]. According to our clinical experience, the success rates of DCR for patients with and without previous episodes of chronic or acute dacryocystitis should be similar.

Our current study presents a series of patients with or without previous episodes of dacryocystitis who underwent external DCR for NLDO and compares their preoperative characteristics, postoperative outcomes, and surgical success rates.

## 2. Methods

Institutional review board (IRB) approval for conducting this study was granted by the Tel Aviv Sourasky Medical Center. We retrospectively reviewed the medical files of all patients who were referred to our tertiary referral center and underwent external DCR between 2006 and 2011. All preoperative evaluations and operations were performed by four experienced surgeons.

The patients were divided into two groups: patients with and without previous episodes of chronic or acute dacryocystitis (as determined by physical examination). Acute dacryocystitis was defined by the appearance of pain, erythema, or pus discharge and swelling of the lacrimal sac. Chronic dacryocystitis was defined by the appearance of epiphora and purulent discharge from the punctum. In the presence of dacryocystitis, the pus was drained and antibiotic treatment with oral amoxicillin and clavulanate (875 mg × 2/d) was started. Only after resolution of the acute phase, an external DCR was performed. In some cases, a CT scan was performed. If the main complaint was epiphora, lacrimal syringing was performed followed by external DCR in patients with NLDO. An otolaryngologist was not involved in patients' evaluation or treatment.

The extracted data included patients' demographics, indication for surgery, background diseases (systemic and ocular), duration of epiphora, side of obstruction, previous external DCR, early and late postoperative complications, duration of the stenting tube, and surgical outcome.

Patients aged at least 16 years with complete NLDO on syringing were recruited. They all underwent external DCR and had a complete postoperative followup of at least 6 months. Excluded were patients who underwent endoscopic DCR, those with a functional obstruction, those aged less than 16 years, and those with inadequate followup. Success was determined by postoperative patency on syringing and resolution of epiphora at 6 months after surgery.

External DCR was performed under general anesthesia. We use a standard technique based on a straight incision made medial to the angular vein at the level of the medial canthal ligament. An osteotomy of 1.5–2 × 1.5–2 cm was created, and the lacrimal sac and mucosa were opened to form anterior and posterior flaps. A silicon tube (BICA, 2012 FCI Ophthalmics, Marshfield Hills, MA) was inserted and tied, after which the anterior and posterior flaps are sutured with 6/0 Vicryl sutures. The wound is closed with absorbable sutures (5/0 Vicryl Rapide or 6/0 Coated Vicryl). All patients were treated postoperatively with topical Maxitrol (dexamethasone, polymyxin B sulphate, and neomycin sulphate) ointment on the skin incision and Ofloxacin eye drops 3 times a day for 7–10 days. If dacryocystitis was evident during the surgery (pus in the lacrimal sac), treatment with oral amoxicillin and clavulanate (875 mg × 2/d) for 5 days was added. The silicon tubes were kept in situ for 6–16 weeks. The removal of the silicone tube took place only when there was a patency on syringing and resolution of epiphora and at least 6 weeks passed from the surgery.

Statistical analysis was performed using Excel and SPSS. The selected variables were compared between the two patient groups. Fisher's exact test and *χ* ^2^ analysis were used for comparison between categorical variables, and a *t*-test was used for comparing continuous variables. For multivariate analyses, a logistic regression was used to calculate the odds ratio (OR) which was used to predict the odds of developing dacryocystitis in the presence of glaucoma or past cataract surgery. A *P* value of 0.05 was considered significant.

## 3. Results

During the study period, 185 patients underwent external DCR and 152 of them met the inclusion criteria. The participants included 100 females and 52 males (mean age 67 ± 15 years), of whom 60 were diagnosed with dacryocystitis (acute or chronic) and 92 were not. The two groups were matched for age (*P* = 0.203). The mean duration of epiphora prior to the first admission to the clinic was 33 months for patients without dacryocystitis and 28 months for patients with a previous episode of dacryocystitis (*P* = 0.001). There was no difference in the percentage of laterality of the obstruction in the former group (27 (29.3%) right, 29 (31.5%) left, and 36 (39.1%) bilateral). In contrast, significantly fewer patients with a previous episode of dacryocystitis had bilateral obstruction (12 (20%), *P* = 0.028), and significantly more of them had a left-side obstruction (29 (48.3%), *P* = 0.031) compared to the patients without dacryocystitis (Table 1).

There was no significant association between hypertension, ischemic heart disease, hyperlipidemia, diabetes, hyperthyroidism, and anemia and the occurrence of dacryocystitis. Two (2.2%) of the 92 patients without a previous episode of dacryocystitis were diagnosed with glaucoma, compared to 10 (16.7%) of the 60 patients with dacryocystitis (*P* = 0.002). The presence of glaucoma with epiphora duration of less than 11.5 months was significantly predictive of the development of dacryocystitis (*P* = 0.019); the odds were 6.71 times greater than those for nonglaucomatous patients. Fourteen patients without dacryocystitis (15.2%) and 17 patients with a previous episode of dacryocystitis (28.3%) underwent cataract surgery in the past, and they had a tendency to develop dacryocystitis in the presence of NLDO (*P* = 0.064) (Table 2).

The mean postoperative follow-up time was 19 months for patients without dacryocystitis and 20 months for patients with a previous episode of dacryocystitis (*P* = 0.783). Early postoperative nasal bleeding was defined as bleeding occurring during the first postoperative day. It occurred in 6 patients without dacryocystitis (6.5%) and in 6 patients with a previous episode of dacryocystitis (10%) (*P* = 0.542). It was mild and stopped spontaneously after several hours in all cases. The silicone tube was removed after a mean duration of 86 days in patients without dacryocystitis and after a mean duration of 79 days in patients with a previous episode of dacryocystitis (*P* = 0.333). After a minimal period of 6 months, patency on syringing and resolution of epiphora was documented in 80 patients without dacryocystitis (86.7%) and in 56 patients with previous episode of dacryocystitis (94.4%) (*P* = 0.337).

## 4. Discussion

Our comparisons of patients' characteristics and surgical outcome results of external DCR in patients with NLDO, with and without a past history of dacryocystitis, revealed no significant differences between the two groups. In the literature, the reported surgical success rates of external DCR in patients with no prior episodes of dacryocystitis ranged from 80% to 96% [3–11]. Our surgical success rate for patients without dacryocystitis was 86.7%.

Only a few earlier studies examined the effect of past dacryocystitis on the success rates of external DCR and reported them as being lower than in cases without dacryocystitis. Badhu et al. [12] follow-up assessments of 662 patients with chronic dacryocystitis who underwent external DCR included symptom evaluation and irrigation of the lacrimal passage. Surgical success was defined by being asymptomatic and having patent lacrimal passages, and it was achieved in 88.6% of their patients. Yigit et al. [13] presented 55 patients with chronic dacryocystitis who underwent external DCR. Successful outcomes were defined as diminished epiphora or no observable reflux from the canaliculus during or after lacrimal irrigation. Success was achieved in 69.9% of their patients. The patients of both studies were followed up at 1, 3, and 6 months and one year after surgery. Additional predictive factors for lower external DCR success rates are the duration of obstruction and patient's age. Erdöl et al. [2] noted that lower success rates are common in younger patients compared to older ones, while early operations offer greater success. Seider et al. [5] found that patients who had longer-lasting symptoms of chronic dacryocystitis had lower success rates.

We found that, in patients with previous episodes of dacryocystitis, the obstruction was more common on the left side. Similar findings were reported in other studies. A suggested explanation is that the angle formed between the left nasolacrimal sac and duct is smaller than that on the right side, and this allows proliferation of bacteria [14, 15].

We found that a shorter mean duration of epiphora (equivalent to a shorter duration of NLDO) correlated with previous episodes of dacryocystitis and that there was no difference in the surgical success rates in these patients. We believe that the reason for similar surgical results in both groups is a shorter duration of epiphora in patients with dacryocystitis.

Our results revealed that patients with glaucoma have higher odds (6.71-fold) to develop dacryocystitis in the presence of NLDO compared to patients without glaucoma. To the best of our knowledge, neither a direct correlation between glaucoma and NLDO nor between glaucoma and dacryocystitis has been reported previously. There are, however, a few reports on antihypertensive medications as a cause of NLDO or lacrimal canalicular obstruction. Seider et al. [16] reported that chronic use of timolol-containing topical glaucoma therapy preparations in glaucoma patients is associated with an increased risk of developing NLDO. Kashkouli et al. and McNab [17, 18] demonstrated that the punctum and canaliculus are the main anatomical sites of lacrimal drainage system obstruction associated with topical antihypertensive medications.

Although topical glaucoma therapy can cause NLDO, we found no reports on a correlation between glaucoma/antihypertensive medications and dacryocystitis, nor any correlation between NLDO caused by topical antihypertensive medications and the occurrence of dacryocystitis.

The correlation we observed between past cataract surgery and NLDO with dacryocystitis did not reach a level of significance (*P* = 0.064). We did not find any studies in the literature on such an association. According to our findings, patients with glaucoma or past cataract surgery who have NLDO are prone to develop dacryocystitis. We therefore suggest that these patients be warned about this complication and instructed to seek medical treatment at the first occurrence of epiphora. Early DCR can prevent dacryocystitis.

The main drawback of this study stems from its retrospective design. In addition, the information on the patients' diseases was taken from their medical charts, and the duration of epiphora was self-reported by the patients.

In conclusion, we found no significant difference in the success rates of external DCR in patients with or without a previous episode of dacryocystitis. Our finding that patients with glaucoma and NLDO have a higher risk of developing dacryocystitis should be confirmed in larger-scale studies.

## Figures and Tables

**Table 1:** Patients' demographics and characteristics.

	No past dacryocystitis, *n* (%)	Past dacryocystitis, *n* (%)	Total, *n* (%)	*P* value
Male/female	33 (35.9)/59 (64.1)	19 (31.7)/41 (68.3)	52 (34.2)/100 (65.8)	0.361
Mean age, y	68 ± 17	66 ± 14	67 ± 15	0.203
Mean duration of epiphora	33 months	28 months	31 months	0.001
Side of obstruction: OS/OD/OU	29 (31.5)/27 (29.3)/36 (39.1)	29 (48.3)/19 (31.7)/12 (20)	58 (38.25)/46 (30.3)/48 (31.6)	0.031/0.993/0.028

OS: left eye; OD: right eye; OU: both eyes.

**Table 2:** Comorbidities.

	No past dacryocystitis	Past dacryocystitis	Total	*P* value
Glaucoma, *n*	2 (2.2%)	10 (16.7%)	12 (7.8%)	0.002
Past cataract surgery, *n*	14 (15.2%)	17 (28.3%)	31 (20.3%)	0.064
Hypertension, *n*	48 (52.2%)	27 (45%)	75 (49%)	0.411
Ischemic heart disease, *n*	12 (13%)	13 (21.7%)	25 (16.5%)	0.183
Hyperlipidemia, *n*	41 (44.6%)	22 (36.7%)	63 (41.4%)	0.400
Diabetes mellitus type 2, *n*	22 (23.9%)	15 (25%)	37 (24.3%)	0.513
Hypothyroidism, *n*	8 (8.7%)	5 (8.3%)	13 (8.5%)	0.301
Anemia, *n*	7 (7.6%)	4 (6.7%)	11 (7.2%)	0.548
